# AI-assisted anatomical structure recognition and segmentation via mamba-transformer architecture in abdominal ultrasound images

**DOI:** 10.3389/frai.2025.1618607

**Published:** 2025-07-23

**Authors:** Shih-Fang Chang, Po-Yi Wu, Ming-Chang Tsai, Vincent S. Tseng, Chi-Chih Wang

**Affiliations:** ^1^Information and Communications Research Laboratories, Industrial Technology Research Institute, Hsinchu, Taiwan; ^2^Department of Computer Science, National Yang Ming Chiao Tung University, Hsinchu, Taiwan; ^3^School of Medicine, Chung Shan Medical University, Taichung, Taiwan; ^4^Division of Gastroenterology and Hepatology, Department of Internal Medicine, Chung Shan Medical University Hospital, Taichung, Taiwan

**Keywords:** anatomical structure, image segmentation, abdominal ultrasound, sonography, artificial intelligence, deep learning, transformer, state space models

## Abstract

**Background:**

Abdominal ultrasonography is a primary diagnostic tool for evaluating medical conditions within the abdominal cavity. Accurate determination of the relative locations of intra-abdominal organs and lesions based on anatomical features in ultrasound images is essential in diagnostic sonography. Recognizing and extracting anatomical landmarks facilitates lesion evaluation and enhances diagnostic interpretation. Recent artificial intelligence (AI) segmentation methods employing deep neural networks (DNNs) and transformers encounter computational efficiency challenges to balance the preservation of feature dependencies information with model efficiency, limiting their clinical applicability.

**Methods:**

The anatomical structure recognition framework, MaskHybrid, was developed using a private dataset comprising 34,711 abdominal ultrasound images of 2,063 patients from CSMUH. The dataset included abdominal organs and vascular structures (hepatic vein, inferior vena cava, portal vein, gallbladder, kidney, pancreas, spleen) and liver lesions (hepatic cyst, tumor). MaskHybrid adopted a mamba-transformer hybrid architecture consisting of an evolved backbone network, encoder, and corresponding decoder to capture long-range spatial dependencies and contextual information effectively, demonstrating improved image segmentation capabilities in visual tasks while mitigating the computational burden associated with the transformer-based attention mechanism.

**Results:**

Experiments on the retrospective dataset achieved a mean average precision (mAP) score of 74.13% for anatomical landmarks segmentation in abdominal ultrasound images. Our proposed framework outperformed baselines across most organ and lesion types and effectively segmented challenging anatomical structures. Moreover, MaskHybrid exhibited a significantly shorter inference time (0.120 ± 0.013 s), achieving 2.5 times faster than large-sized AI models of similar size. Combining Mamba and transformer architectures, this hybrid design was well-suited for the timely analysis of complex anatomical structures segmentation in abdominal ultrasonography, where accuracy and efficiency are critical in clinical practice.

**Conclusion:**

The proposed mamba-transformer hybrid recognition framework simultaneously detects and segments multiple abdominal organs and lesions in ultrasound images, achieving superior segmentation accuracy, visualization effect, and inference efficiency, thereby facilitating improved medical image interpretation and near real-time diagnostic sonography that meets clinical needs.

## 1 Introduction

Abdominal ultrasonography (US) is a primary diagnostic tool for evaluating medical conditions within the abdominal cavity and discomfort (Tomizawa et al., 2017). Physicians frequently use the abdominal US to screen for lesions in abdominal organs, including the liver, gallbladder, kidneys, pancreas, spleen, and adjacent blood vessels, facilitating a comprehensive assessment of intra-abdominal structures. The deep location of abdominal organs within the body and their potential obscuration by bone structures or intestinal gas often result in partially captured organ images on abdominal ultrasound. Variations in ultrasound imaging equipment and systems further complicate image interpretation, posing significant assessment challenges. Despite its relatively lower image resolution compared to advanced medical imaging modalities such as computed tomography (CT) and magnetic resonance imaging (MRI), US remains irreplaceable for the timely detection of potentially life-threatening conditions such as acute abdomen and supports further diagnosis and intervention.

Identifying the relative location of abdominal organs and lesions based on anatomical or pathological features in US images is essential in diagnostic sonography. However, due to the inherent characteristics of ultrasound imaging, including blurred textures and indistinct organ boundaries, interpretation can be challenging, particularly for inexperienced physicians and inadequately trained technicians (Reddy et al., 2021). With the rise of artificial intelligence (AI), deep neural network (DNN) techniques (Reddy et al., 2021; Cheng and Malhi, 2017; Dandan et al., 2020; Xu et al., 2018; Hatture and Kadakol, 2021) have shown promise in facilitating object detection and instance segmentation of abdominal organs, reducing examination interpretation time in US images. Due to the time-consuming annotation process, most studies on the abdominal US have trained their AI models using small datasets, typically comprising only hundreds or thousands of labeled images (Song, 2021). Consequently, extracting features from limited data to enhance model training is essential for improving generalizability in clinical applications.

Object detection and instance segmentation in US image analysis aims to identify regions of interest (ROI) as reference points for lesion assessment and aid in diagnostic interpretation. Therefore, accurate ROI extraction is critical for defining organs and lesion boundaries in abdominal US images. Historically, abdominal anatomical recognition heavily relied on the generation of hand-crafted image characteristics to expand feature dimension spaces. For instance, the light neural network, a time-sensitive attention-radial basis function network (TSA-RBFN), was designed to calculate distances within feature dimensions, aiding in segmenting and measuring inflamed gallbladder volumes associated with cholecystitis and gallstones (Muneeswaran and Rajasekaran, 2018). Similarly, wavelet decomposition has been employed in high-resolution US images to enhance gallbladder localization and facilitate the detection of suspicious gallbladder polyps (Chen et al., 2020). Active contour segmentation with wavelet filtering has also been further applied to liver disease classification (Krishnan and Radhakrishnan, 2017). More recently, the continued advancement of deep learning has revolutionized abdominal US imaging applications (Cai and Pfob, 2025), particularly in the automatic feature extraction and recognition of abdominal organs such as the kidney (Ravishankar et al., 2017; Yin et al., 2019; Yin et al., 2020; Peng et al., 2023; Peng et al., 2023), prostate (Peng et al., 2023; Karimi et al., 2019; Lei et al., 2019; Orlando et al., 2020), gallbladder (Obaid et al., 2023), and liver (Ryu et al., 2021; Dadoun et al., 2022; Mămuleanu et al., 2022; Lee et al., 2020; Biswas et al., 2018; Xi et al., 2021; Turco et al., 2022).

Ryu et al. (2021) introduced a multi-task system based on the Visual Geometry Group Network (VGG-Net) for segmenting and classifying liver lesions in US images with user-provided click guidance. Ravishankar et al. (2017) developed a shape-regularized U-Net (SR-UNet) segmentation framework that integrates shape priors into fully convolutional networks to enhance robustness against low contrast and artifacts. Strategies incorporating morphological information have also improved the effectiveness of DNN-based segmentation tasks. Yin et al. (2019) and Yin et al. (2020) developed a boundary distance regression network to improve the segmentation robustness against variations in kidney appearance. Peng et al. employed a contour extraction approach (Peng et al., 2023) and an automatic searching polygon tracking method (Peng et al., 2023) to address the challenges of unclear boundaries and diverse kidney shapes in US images. Similarly, Obaid (Obaid et al., 2023) applied active contour segmentation integrated with DNN models to delineate organ boundaries and classify gallbladder disease. Additionally, blood vessels within the liver are critical anatomical landmarks in delineating the liver’s anatomy and identifying adjacent organs, such as the pancreas. Deep learning (U-Net) and detection transformer (DETR) have been applied to the characteristic identification and lesion segmentation of liver diseases, including hepatic cysts and tumors (Ryu et al., 2021; Dadoun et al., 2022; Mămuleanu et al., 2022). Lee et al. (2020) utilized deep learning (VGG-Net) to predict the meta-analysis of histological data in viral hepatitis (METAVIR) score and classify liver fibrosis severity for screening and longitudinal assessment of US examinations. Biswas et al. (2018) applied a deeper DNN structure (GoogLeNet) to characterize tissue in fatty liver disease and stratify normal and abnormal tissues. Xi et al. (2021) employed a similar DNN architecture (ResNet) with pre-trained weights to distinguish between benign and malignant liver lesions. Advanced supervised multidirectional DNN mechanisms (3D V-Net series) (Lei et al., 2019; Orlando et al., 2020) were further employed to segment prostate volume for prostate cancer diagnostic applications. Despite having relatively shallow architecture with only a few dozen layers, these deep-learning models have outperformed radiologists in specific tasks.

Moreover, Turco et al. (2022) interpreted the spatiotemporal features of vascular perfusion and characterized vascular structures in contrast-enhanced ultrasound (CEUS) to improve the precise characterization of focal liver lesions. Zhang et al. (2024) proposed the SEG-LUS semantic segmentation model, incorporating multi-head self-attention to identify small ROIs, such as the inferior vena cava, portal vein branches, and hepatic artery, during clinical scanning. While current studies have reported the dice scores (0.826–0.957; Ravishankar et al., 2017; Peng et al., 2023; Peng et al., 2023; Zhang et al., 2024) and diagnostic accuracies (83.5–98.4%; Peng et al., 2023; Obaid et al., 2023; Lee et al., 2020) for abdominal US, most research focuses on learning from US images with a single label per image in multi-organ scenes and subsequently inferring the organ with the highest probability. Such limitations hinder the applicability of these methods in clinical scenarios that require the simultaneous identification of multiple organs to enhance the diagnostic quality in abdominal ultrasound imaging.

This study aimed to develop an efficient AI-based anatomical recognition framework capable of automatically and simultaneously detecting and segmenting multiple anatomical landmarks from the abdominal US images, thereby enhancing generalizability and diagnostic accuracy in clinical practice.

## 2 Materials and methods

### 2.1 Study population

#### 2.1.1 Participants

The retrospective dataset was obtained from outpatient examinations using ultrasound scanners from Toshiba, Hitachi, General Electric, Canon, and Siemens at Chung Shan Medical University Hospital (CSMUH) between April 2013 and May 2024. It included 34,711 B-type abdominal ultrasound images (format: JPG, size: 480 × 640 to 970 × 1,552 pixels) from 2,063 patients (male: 56.8%, female: 43.2%). This study underwent a medical ethics review and was approved by the CSMUH Institutional Review Board (IRB) (IRB No: CS2-22003), and all patient identities were anonymized before images were released, eliminating the need for informed consent from included patients.

#### 2.1.2 Data annotation

**Figure 1:**
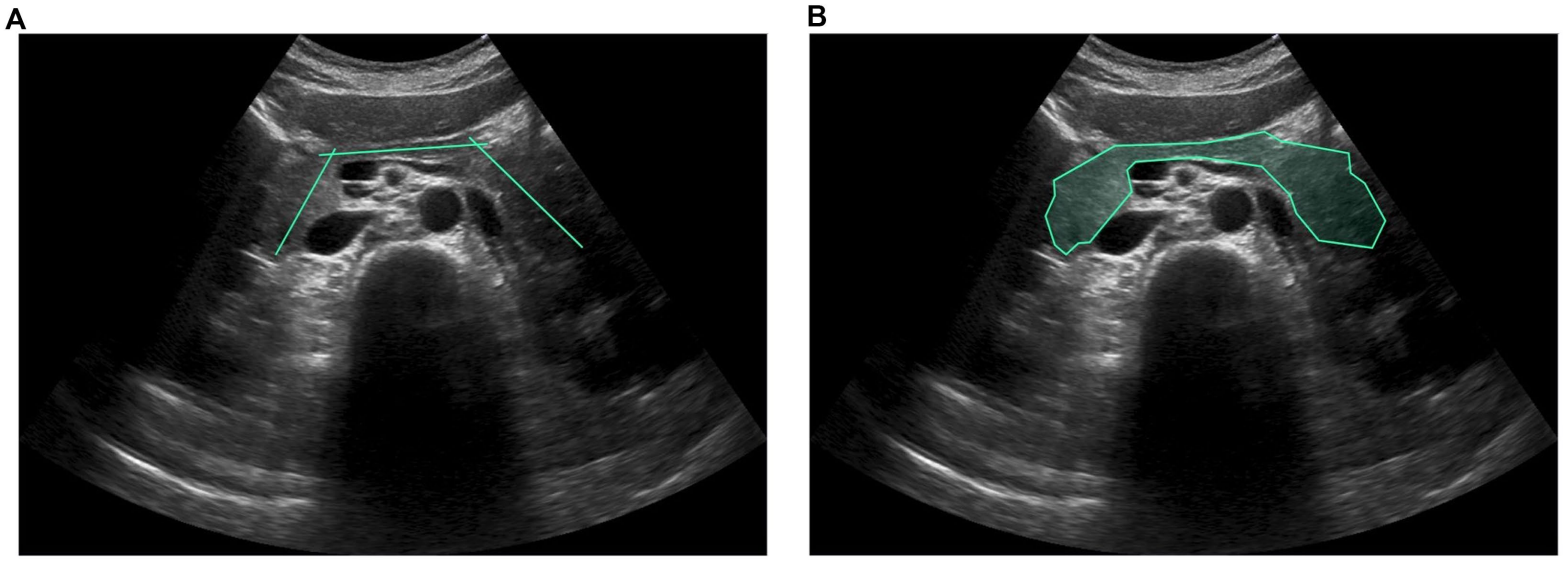
**(A)** Line segments and **(B)** polygon-based contouring foregrounds were created with our own interactive labeling mechanism. Intersecting line segments annotated by physicians are used to indicate potential organ regions, and the GrabCut segmentation algorithm is then used to generate the ground truth automatically.

Given that the quality of collected US images can be affected by various health conditions of the abdominal organs, several internists specializing in hepatology and gastroenterology (with 8–20 years of professional experience) were invited to establish accurate annotations for a high-quality dataset. The dataset was annotated using an interactive labeling mechanism for image segmentation to facilitate AI-assisted recognition of organs and related lesions required for US examination, thereby reducing labor-intensive processes. After each US image set of the same patient case was randomly assigned to a physician, the physicians marked only the intersecting line segments to indicate potential organ regions. A polygon-based contouring foreground was then automatically generated using the optimized graph-based segmentation algorithm, GrabCut (Rother et al., 2004; Xu et al., 2017), which progressively enhances and streamlines the segmentation contour endpoints by considering color resemblance and spatial closeness, as illustrated in Figure 1. The ground truth was then established for the dataset for subsequent modeling of detection and segmentation tasks. This dataset involved seven abdominal organs and vascular structures, including the hepatic vein, inferior vena cava, portal vein, gallbladder, kidney, pancreas, and spleen, and two related liver lesions, including the hepatic cysts and tumors, comprising 6,332, 3,977, 16,202, 8,183, 5,858, 3,492, 1,358, 2,630, and 8,191 marks, respectively.

#### 2.1.3 Dataset split

**Figure 2:**
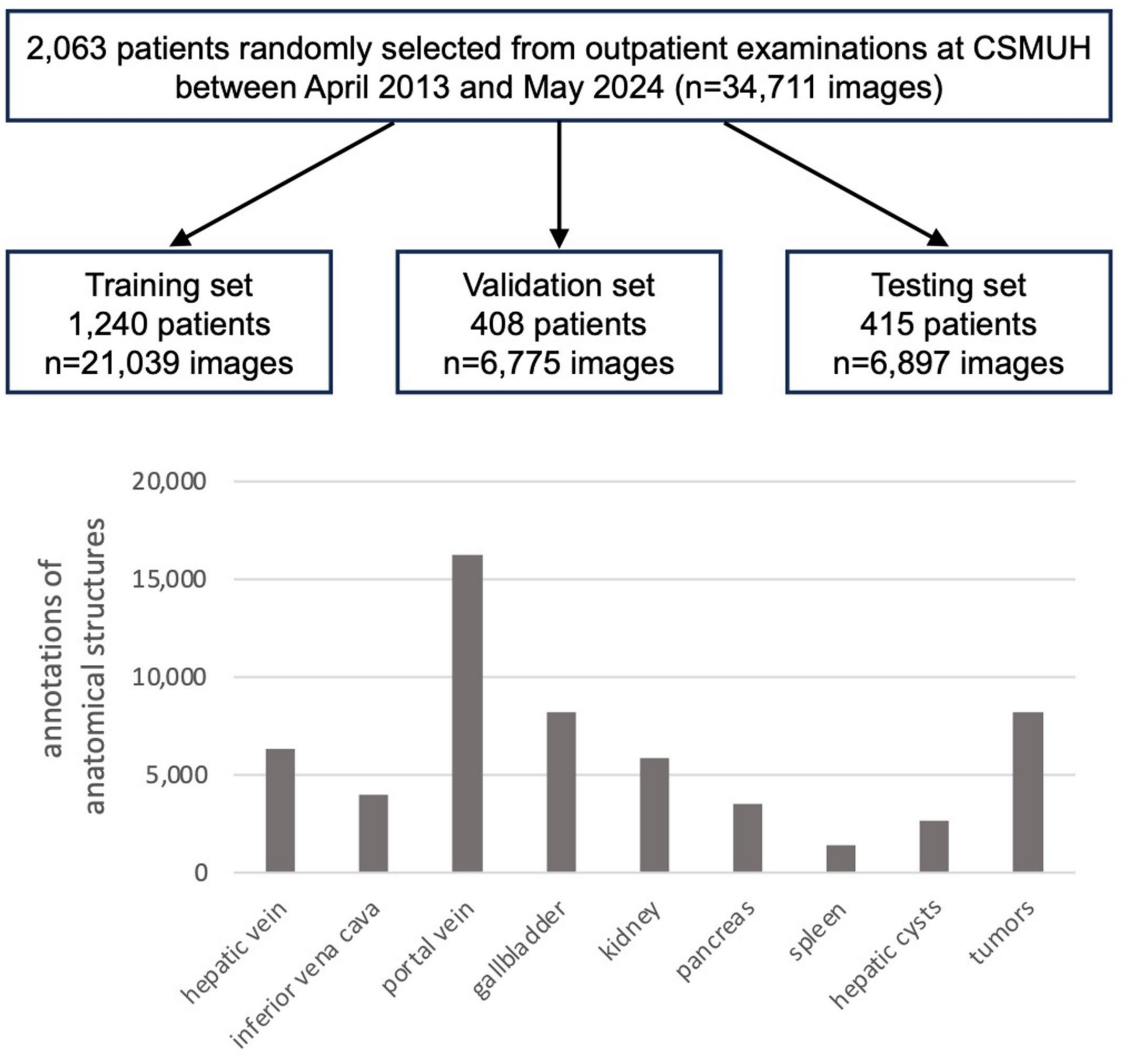
Number of patients and ultrasound images, and the distribution of anatomical structure annotations in the private dataset.

The entire private dataset of patients and US images was randomly divided into training, validation, and testing sets based on patient cases, comprising 21,039, 6,775, and 6,897 images of 1,240, 408, and 415 cases, respectively, with approximate ratios of 60, 20, and 20%. Table 1 and Figure 2 present detailed information regarding the number of images and the distribution of annotations for anatomical structures.

**Table 1:** The training, validation, and testing sub-datasets for abdominal anatomical recognition modeling.

Anatomical Landmarks	Training		Validation		Testing		Total		
	cases	images	cases	images	cases	images	cases	images	marks
Hepatic vein	791	2,878	247	970	258	926	1,296	4,774	6,332
Inferior vena cava	673	2,340	220	827	224	805	1,117	3,972	3,977
Portal vein	1,100	7,893	352	2,561	366	2,586	1,818	13,040	16,202
Gallbladder	984	4,894	329	1,558	328	1,593	1,641	8,045	8,183
Kidney	1,034	3,564	346	1,149	343	1,144	1,723	5,857	5,858
Pancreas	806	2,098	265	662	265	730	1,336	3,490	3,492
Spleen	536	810	177	274	182	273	895	1,357	1,358
Hepatic cyst	382	1,421	130	408	119	384	631	2,213	2,630
Tumor	566	3,727	186	1,271	185	1,260	937	6,258	8,191
Total	1,240	21,039	408	6,775	415	6,897	2,063	34,711	56,223

Each ultrasound image set of the same patient case will be assigned to only one of the training, validation, and testing sub-datasets.

### 2.2 Studies for algorithm development

In the pre-deep learning era, image segmentation for anatomical landmark recognition relied on hand-crafted features and classical computer vision techniques, which were limited by occlusion and overlapping objects. Currently, the use of DNNs and transformers to enhance segmentation accuracy and scalability represents one of the main trends in contemporary medical image analysis.

#### 2.2.1 DNN-based segmentation

Mask R-CNN (He et al., 2017) is a typical two-stage image segmentation design (object detection followed by pixel-by-pixel mask prediction) that deals with varying shapes and generalizes to several types of scenes, handling simple overlapping objects effectively. The YOLO (You Only Look Once) series (Boesch, 2024) employs a single-stage detection paradigm and has evolved significantly in speed, accuracy, feature extraction, and computational efficiency. It evolved from its grid-based, single-stage detection (YOLOv1) to a multi-scale anchor-based approach (YOLOv2, YOLOv3), improving accuracy and robustness. CSPDarkNet, PANet, CIoU loss (YOLOv4), and anchor-free designs with model scaling (YOLOv6, YOLOv7) optimized speed and precision. PGI, GELAN (YOLOv9), and the removal of Non-Maximum Suppression (YOLOv10) enhanced detection and segmentation speed. The latest version, YOLOv11, integrates object detection, segmentation, pose estimation, oriented bounding boxes, and classification to advance performance. However, the trade-off between high-precision segmentation and computational efficiency in DNN-based segmentation limits its applicability in real-world scenarios (Xu et al., 2024).

#### 2.2.2 Transformer-based segmentation

Recent advancements have used transformers for image segmentation, improving the understanding of the global context. The architecture consists of an encoder-decoder structure, where the encoder captures contextual information and the decoder generates the output sequence. The self-attention mechanism enables transformers to evaluate the relative importance of input elements, effectively capturing long-range dependencies that may be overlooked by recurrent neural networks (RNNs) and convolutional neural networks (CNNs). This capability is particularly beneficial for segmental tasks, where the relevant context may not be confined to regions locally in US images. DETR (Carion et al., 2020) was the first transformer to use query embeddings, combining CNN feature extraction with transformer-based decoding for object detection and instance segmentation, achieving end-to-end but at a high computational cost. By incorporating query embedding refinements and enhanced attention mechanisms, DINO (DETR with improved deNoising anchOrs) (Zhang et al., 2023) stabilized bipartite matching and contrastive query selection, enhancing feature learning and convergence speed in detection and segmentation tasks. This approach accelerated learning while maintaining high precision, forming the foundation for models like MaskDINO (Li et al., 2023) for dense prediction tasks. MaskDINO integrated DETR-like object detection and the DINO structure with mask prediction capabilities to extend efficient semantic and panoptic segmentation advantages. With multi-scale features, self-attention, and contrastive denoising training, MaskDINO captured global dependencies, leading to improved accuracy and robustness compared to DNN-based approaches. However, these transformer-based segmentation approaches still encounter computational efficiency challenges, hindering their applicability in real-time applications without optimization.

#### 2.2.3 State space models (SSM)

SSM is a mathematical framework that models sequence or time series data by maintaining a hidden internal state that evolves based on input signals and past states, optimizing model inference speed while maintaining model effectiveness. SSM is widely used in signal processing, control systems, time series forecasting, and deep learning. Mamba (Gu and Dao, 2024), a modern SSM-based sequence model, was introduced with gated state transitions to enhance expressiveness while maintaining efficiency. It features parallelizable recurrence, reducing memory overhead and improving long-sequence modeling. By leveraging input-dependent gating and efficient kernel parameterization, Mamba achieves transformer-level performance while being computationally efficient for NLP and vision tasks. Mamba-2 (Dao and Gu, 2024) built upon Mamba, refining its gating mechanisms, adaptive state transitions, parallelized recurrence, and efficient parameterization, achieving better expressiveness, efficiency, and scalability for long-range dependency modeling and rivaling transformers while reducing computational overhead. On the other hand, MambaVision (Hatamizadeh and Kautz, 2024) proposed a hybrid mamba-transformer backbone, adopting Mamba for vision tasks and offering an effective alternative to deep learning and transformers for image and video understanding.

### 2.3 MaskHybrid, the proposed framework

**Figure 3:**
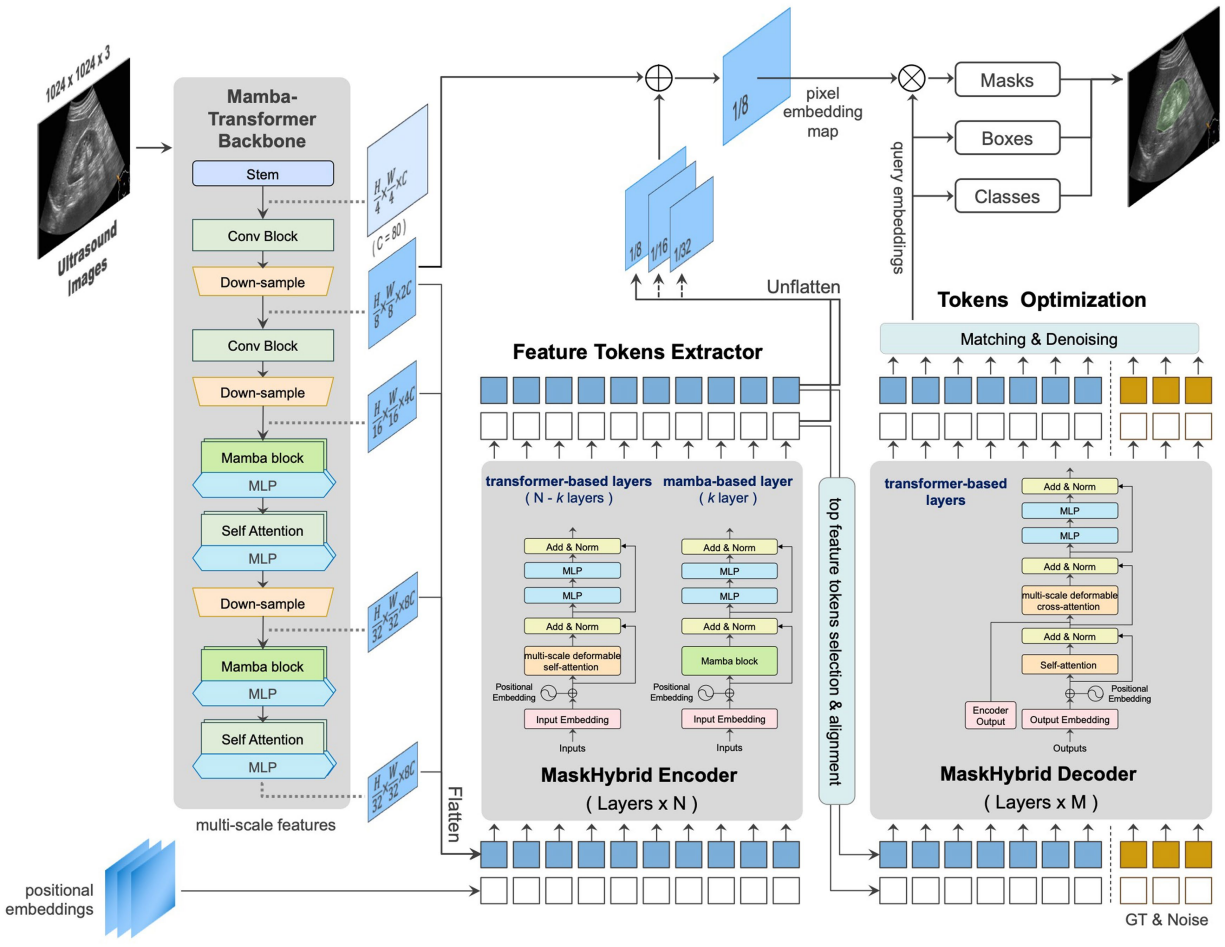
Architecture and components of the proposed framework, MaskHybrid, are based on MaskDINO and further extended (gray-shaded area) to accelerate segmentation accuracy and inference efficiency.

We developed an AI-based anatomical recognition framework that presents the mamba-transformer hybrid design to enhance segmentation accuracy, visualization effects, and inference efficiency while mitigating the computational burden. Figure 3 illustrates the architecture of the proposed framework and its major components: the mamba-transformer backbone, the MaskHybrid hybrid encoder, and the corresponding decoder. The hybrid designs were primarily implemented at the backbone and encoder levels to enhance the model performance and visualization for anatomical recognition.

#### 2.3.1 Mamba-transformer backbone

In order to capture global dependencies while preserving the spatial structure of US images, the MambaVision-like architecture was employed to extract multi-scale features across different dimensions. The first two stages retained residual convolutional blocks for rapid feature extraction, while the subsequent two stages were modified to incorporate four layers of the Mamba-2-like blocks and followed by four layers of multi-head self-attention transformer blocks. This design preserves global context and long-range spatial dependencies in vision tasks, as shown in Supplementary Figure 1. At this stage, features were flattened and transformed at different scales for use in the subsequent hybrid encoder.

#### 2.3.2 MaskHybrid encoder

The hybrid encoder consisted of multiple repeated transformer-based encoder layers (*N* = 6), each containing a multi-scale deformable self-attention block and two multilayer perceptron (MLP) blocks. Given the efficacy of Mamba designs in capturing long-range dependencies with a limited number of layers (Dao and Gu, 2024), one of the intermediate layers was replaced by a Mamba-based layer, incorporating a Mamba-2-like block to substitute the self-attention process inherent in the transformer layer. The reference architecture of the encoder layers is shown in Supplementary Figure 2.

#### 2.3.3 MaskHybrid decoder

Unlike the encoder, which incorporated a mamba-based layer, the decoder comprised only multiple repeated transformer-based decoder layers (M = 9), with each decoder layer including an additional multi-head cross-attention compared to the encoder layer. For small-token counts, such as feature tokens derived from our proposed encoder, the Mamba design using recurrent-like state-space updates may exhibit suboptimal learning due to its reliance on hidden state updates rather than pairwise token interactions. This limitation could reduce effectiveness due to weaker token-to-token interactions than transformers (Dao and Gu, 2024). Therefore, the decoder was designed exclusively using transformer-based layers. The reference architecture of the decoder layers is shown in Supplementary Figure 3.

### 2.4 Performance evaluation

Average Precision (AP) is a performance metric used to measure the model capabilities, commonly employed in computer vision tasks like object detection and instance segmentation. In specific applications, AP can be further categorized based on the task at hand, such as box AP and mask AP. Box AP focuses on the overlap between the predicted bounding box and the ground truth box at a fixed intersection over union (IoU) threshold, calculating the area under the precision-recall curve (PR-AUC) by varying the confidence level. Mask AP (He et al., 2017) is specifically designed explicitly for image segmentation tasks, emphasizing the overlap between the prediction and the ground truth masks at a specified IoU threshold. In this study, mask AP was used as the performance metric, and the mAP was then calculated as the mean of the mask AP values across all organ and lesion classes of abdominal anatomical landmarks, with the IoU threshold set to 0.15 and the confidence level set to 0.3, denoted as mAP15. Since a higher IoU threshold (such as IoU ≥ 0.5) may lead to mistakenly excluding clinically reasonable predictions, a lower IoU threshold of 0.15 was determined for model training to appropriately mark anatomical structures and pathological features in a practical clinical setting.

$$\mathrm{IoU}=\frac{\text{Area of Overlap}(\text{prediction mask},\text{ground truth mask})}{\text{Area of Union}(\text{prediction mask},\text{ground truth mask})}$$

$$AP_{\text{class}}=\sum_{i}(r_{i}-r_{i-1})P_{\text{interp}}(r_{i})$$

Where *P_interp_*(*r*) is the interpolated precision at a certain recall level *r*, which is defined as the highest precision found for any recall level *r’* ≥ *r*.

$$\mathrm{mAP}=\frac{1}{n}\sum_{\text{classes}}AP_{\text{class}}$$

Where *AP_class_* is the average precision of each class, and n is the number of classes.

### 2.5 Implementation detail

In this study, original US images were uniformly resized to a dimension of 1,024 × 1,024 pixels, subjected to random horizontal flipping, and augmented with slight scaling using large-scale jittering (LSJ) (Ghiasi et al., 2021) prior to training. The ResNet-50 (He et al., 2016) and Swin Transformer (Swin-T) (Liu et al., 2021) were used as backbones for baseline establishment within MaskDINO, representing small and large-sized models, respectively. The MaskHybrid model utilized six encoder layers and nine decoder layers (*N* = 6, M = 9). The feature channels in both the encoder and decoder were maintained at 256, and the hidden dimension of the feed-forward neural network (FFN) was set to 2,048. The same loss functions as MaskDINO (L1 loss and GIOU loss for box loss, focal loss for classification loss, and cross-entropy loss and dice loss for mask loss) were leveraged for model convergence. Unlike the commonly used IoU threshold of 0.5 for balanced evaluation on public datasets, a threshold of 0.15 (Wang et al., 2022) was chosen because some anatomical landmarks had not been fully annotated by experts, resulting in the actual annotated size being relatively small compared to the ground truth. Employing a lower threshold may reduce the number of false negatives and affect overall model performance; however, it is considered more appropriate for screening purposes in clinical settings.

The fixed input image size results in a constrained token size given by the backbone network to the subsequent encoder and decoder. The tokens of MaskDINO (ResNet), MaskDINO (Swin-T), and MaskHybrid models are 21,760, 21,760, and 22,528, respectively. Since these token sizes are comparable, the candidate models require similar computing resources, with no significant impact on execution time or speed. In addition, due to hardware constraints, the baseline MaskDINO and our MaskHybrid were trained for 10 epochs on an NVIDIA RTX A6000 GPU with an initial learning rate of 1e-4. A batch size of two was used for evaluation to ensure a fair comparison, given the high memory consumption of the Swin-T backbone. An early stopping mechanism was implemented to prevent overfitting. All experiments were conducted using the PyTorch framework in this study.

## 3 Results

### 3.1 Main results of anatomical recognition

As presented in Table 2, the experiments demonstrated the efficacy of incorporating mamba-transformer architectures in anatomical landmarks segmentation. Specifically, the MaskHybrid model outperformed MaskDINO baselines with ResNet-50 and Swin Transformer backbones across most abdominal organs and lesion types, achieving a superior mAP15 score (74.13% vs. 70.68 and 72.60%). For example, it achieved the highest AP scores for the gallbladder (91.79%), kidney (95.47%), pancreas (89.36%), spleen (86.19%), hepatic vein (60.71%) and hepatic cyst (55.48%), suggesting that our model excels at detecting both organs and vascular structures. It also showed consistent segmentation performance, achieving 40.94% mAP at an average AP of higher IoU thresholds from 0.5 to 0.95, surpassing the baselines MaskDINO (Swin-T and ResNet), as shown in Supplementary Table 1. Since this study aims to provide a screening-based toolkit to assist inexperienced physicians or in the medical settings of rural areas, a lower IoU threshold was adopted to better recognize anatomical structures and pathological features, despite performing relatively well at most IoU thresholds.

**Table 2:** Image segmentation performance of MaskHybrid under mAP15 metric compared to MaskDINO baselines with RestNet-50 and Swin Transformer backbones.

Models	Dataset	mAP15 (%)	Average Precision (%)								
			Hepatic vein	Inferior vena cava	Portal vein	Gall-bladder	Kidney	Pancreas	Spleen	Hepatic cyst	Tumor
MaskDINO (ResNet)	valid	70.85	59.92	56.77	66.89	88.66	95.77	88.23	83.91	47.03	50.49
	test	70.68	57.63	63.61	64.38	88.85	94.44	86.30	82.29	51.46	47.15
MaskDINO (Swin-T)	valid	72.59	60.13	**61.68**	69.50	90.01	96.71	89.54	83.19	47.43	**55.12**
	test	72.60	55.24	**67.13**	68.29	91.58	95.29	85.47	83.74	53.27	**53.39**
MaskHybrid	valid	**73.72**	**64.35**	60.36	**71.05**	**90.63**	**97.79**	**91.64**	**87.58**	**48.55**	51.56
	test	**74.13**	**60.71**	66.87	**70.67**	**91.79**	**95.47**	**89.36**	**86.19**	**55.48**	50.62

The MaskHybrid framework improved the overall segmentation performance, reaching 74.13% mAP, surpassing the baselines (70.68–72.60%). Compared to large-sized models of similar size, the MaskHybrid demonstrated comparable segmentation capabilities to the transformer-based object detection framework, MaskDINO (Swin-T), and it also had superior recognition ability and extensibility to small-sized model, MaskDINO (ResNet). The bold values represent the best-performing results among the models.

The performance improvement is likely due to the enhanced contextual modeling provided by the mamba-transformer architecture, which supports longer-range dependencies and improved spatial reasoning. Furthermore, the MaskHybrid model showed significant improvements in segmenting challenging anatomical structures such as the hepatic vein, portal vein, and hepatic cyst. Larger organs (including the gallbladder, kidneys, pancreas, and spleen: 86.19–95.47%) exhibited higher AP than blood vessels (60.71–70.67%) due to their relatively larger volumes. These findings suggested that the mamba-transformer hybrid design effectively captured long-range spatial dependencies and contextual information, making it well-suited for complex ultrasound image segmentation tasks where accuracy and robustness are critical.

**Figure 4:**
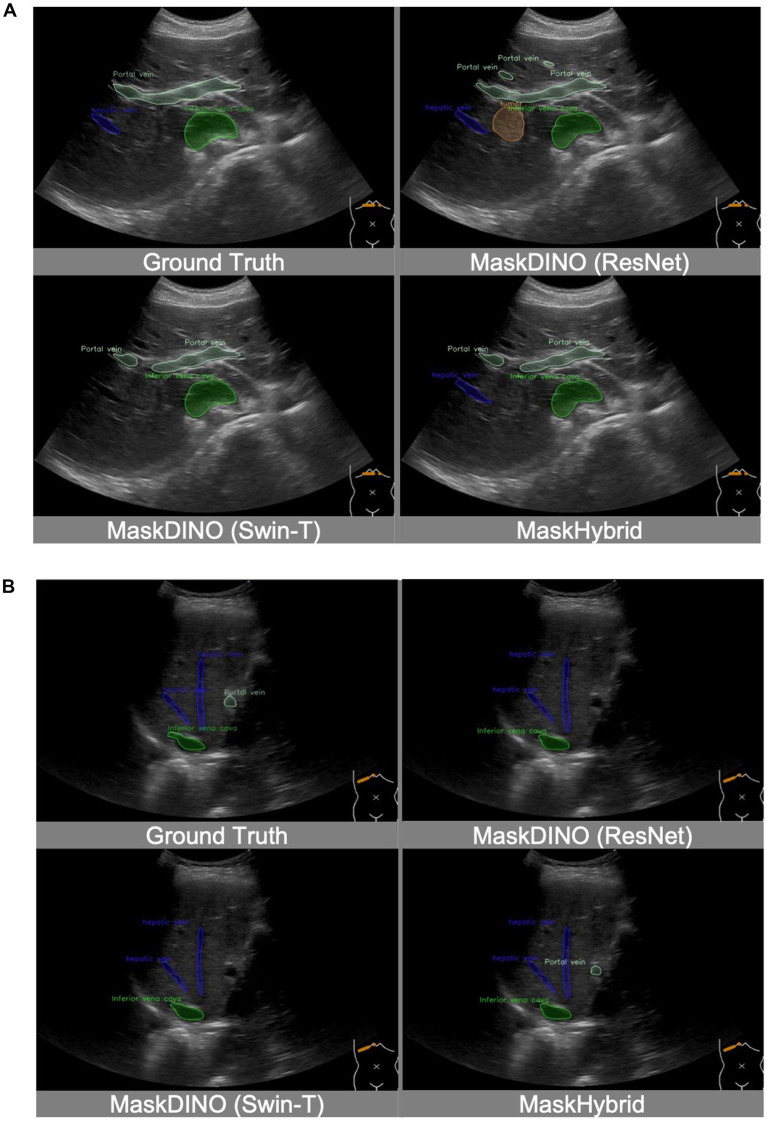
Visualization comparison of baselines and our anatomical recognition model. MaskHybrid achieved the closest visualization effect to the ground truth regarding both annotation type and the number of recognized structures. **(A)** MaskDINO baselines missed the hepatic vein or the erroneous identification of the tumor. **(B)** MaskDINO baselines missed the portal vein.

In addition, the MaskHybrid model, incorporating the mamba-transformer hybrid design, achieved the closest visualization effect to the ground truth regarding both annotation type and the number of recognized structures. In contrast, the MaskDINO baselines exhibited missed anatomical structures (hepatic vein in Figure 4A and portal vein in Figure 4B) or the erroneous identification of non-existent lesions (tumor in Figure 4A). However, these structures were correctly recognized in our MaskHybird model. Detailed comparisons between models are provided in the Supplementary Material.

### 3.2 Ablation experiments

To further investigate the effect of the mamba-transformer hybrid design, ablation experiments were performed on the segmentation performance of anatomical structures with various backbone and encoder combinations. As presented in Table 3, the hybrid architecture of MaskHybrid (Mamba-T) as the backbone with MaskDINO as the encoder achieved superior performance, yielding the highest mAP score of 74.13% among all configurations. This indicates that the hybrid architecture, particularly the Mamba-based backbone, is effective in capturing complex anatomical features and enhancing overall model accuracy. Specifically, replacing the Swin-T backbone in the baseline with our mamba-transformer architecture (Mamba-T) improved overall performance, demonstrating superior segmentation accuracy for nearly all anatomical structures.

**Table 3:** Performance variation of different backbone and encoder combinations.

backbone	encoder	mAP15 (%)	Average Precision (%)								
			Hepatic vein	Inferior vena cava	Portal vein	Gall-bladder	Kidney	Pancreas	Spleen	Hepatic cyst	Tumor
MaskDINO (ResNet)	MaskDINO	70.68	57.63	63.61	64.38	88.85	94.44	86.30	82.29	51.46	47.15
MaskDINO (Swin-T)	MaskDINO	72.60	55.24	**67.13**	68.29	91.58	95.29	85.47	83.74	53.27	**53.39**
MaskHybrid (Mamba-T)	MaskDINO	**74.13**	**60.71**	66.87	70.67	**91.79**	**95.47**	**89.36**	**86.19**	**55.48**	50.62
MaskHybrid (Mamba-T)	MaskHybrid	73.63	60.24	65.93	**70.92**	91.64	95.43	87.92	84.27	54.51	51.81

The hybrid architecture with the mamba-transformer backbone achieved the highest model performance. The additional use of a MaskHybrid encoder can provide better visual explanations in specific clinical scenarios while still maintaining competitive performance. The bold values represent the best-performing results among the models.

Notably, the MaskHybrid backbone paired with the MaskHybrid encoder achieved the highest AP for the Portal vein (70.92%), suggesting that matching the backbone and encoder architecture might lead to better feature alignment and precision for certain structures. On the other hand, while the MaskDINO (Swin-T) backbone and MaskDINO encoder pair had a slightly lower mAP (72.60%), it produced the highest AP for the inferior vena cava (67.13%) and tumor (53.39%), entailing that attention-based models may still offer specific benefits for difficult or irregular structure regions. In contrast, the MaskDINO (ResNet) backbone and MaskDINO encoder pair showed the lowest overall performance, both in terms of mAP and per-class AP scores. This underperformance revealed the limitations of early DNN-based backbones such as ResNet in comparison to transformer-based and derivative models in performing complex medical segmentation tasks.

**Figure 5:**
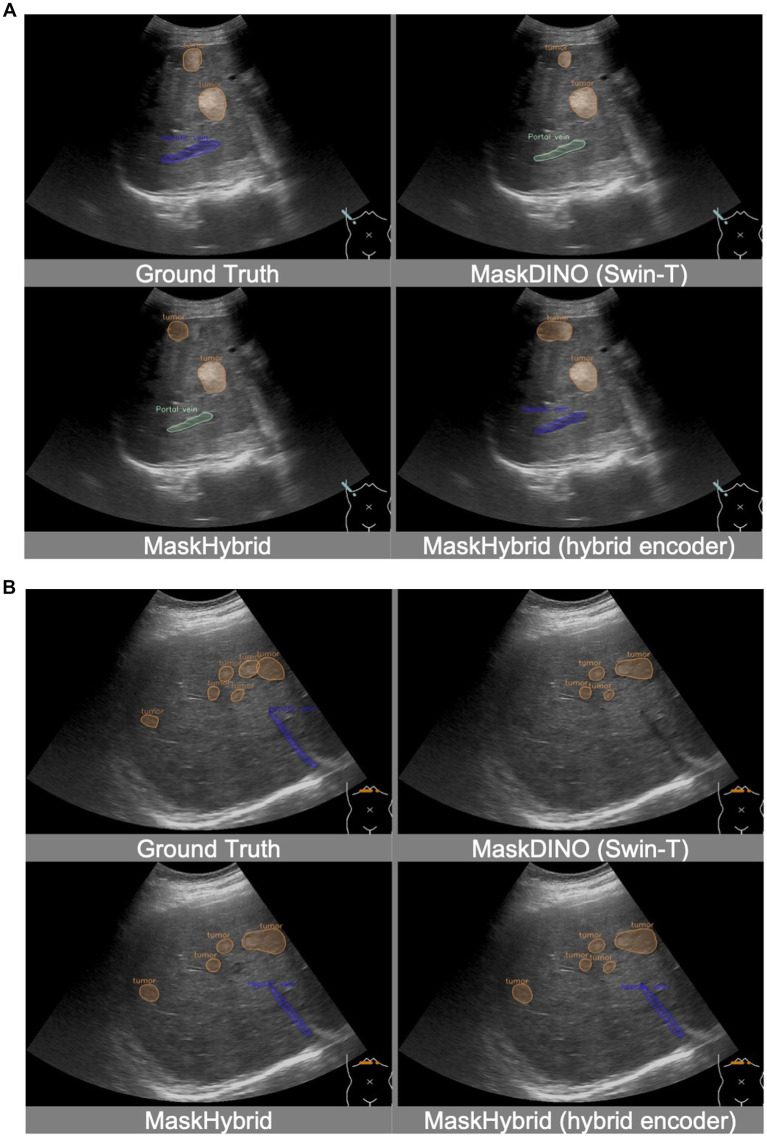
Visualization comparison of MaskHybrid models with and without the hybrid encoder. Both **(A)** and **(B)** are segmentations of hepatic veins and tumors. MaskHybrid with the hybrid encoder correctly identified the hepatic vein in **(A)** and showed a more comprehensive tumor distribution in **(B)**.

Additionally, incorporating the MaskHybrid encoder enhanced visual interpretation in some clinical scenarios while still maintaining similar competitive performance, validating the effectiveness of this novel approach. For instance, while candidate models approximated the location of anatomical structures, only the MaskHybrid with hybrid encoder correctly identified the hepatic vein in Figure 5A and showed a more comprehensive tumor distribution than MaskDINO (Swin-T) and MaskHybrid in Figure 5B.

## 4 Discussion

In contemporary AI development, model accuracy remains a primary objective across all learning tasks. Driven by advancements in deep learning technologies, medical AI research frequently incorporates deeper and more complex network structures to demonstrate the capability to recognize anatomical structures comprehensively, which may sacrifice recognition efficiency.

### 4.1 Computational efficiency

Transformer-based models enhance accuracy through attention mechanisms that selectively focus on critical information. However, the computational complexity of the attention mechanism increases substantially with the number of features, rendering it computationally inefficient and challenging for real-time clinical applications. Given that practical AI applications frequently require timely responses, posing a challenge to balancing accuracy and efficiency, we utilized a hybrid design of Mamba and transformer architectures. This approach led to the development of an enhanced AI detection and segmentation framework, MaskHybrid, aimed at reducing inference latency while preserving model performance advantages. Previous studies (Dao and Gu, 2024)–(Hatamizadeh and Kautz, 2024) have shown that hybrid architectures incorporating a limited number of Mamba layers alongside attention layers can achieve state-of-the-art evaluation metrics and visual representation. Consequently, we facilitated the hybrid design by modifying the model at the backbone and encoder levels to enhance the performance of the anatomical recognition model.

### 4.2 Visualization effect

Visualizing image segmentation of intra-abdominal organs can be challenging due to disease symptoms. For example, large tumor areas can lead to poor organ recognition performance in segmentation models, as seen with the hepatic vein of ground truth in Supplementary Figure 6B. Although images may be affected by associated lesions, the baseline MaskDINO and our MaskHybrid models performed well, accurately delineating tumor regions closest to the ground truth. Furthermore, MaskHybrid mitigated the issue of overlapping segmented regions in identical anatomical structures, resulting in superior overall visualization outcomes.

### 4.3 Modeling limitation

**Figure 6:**
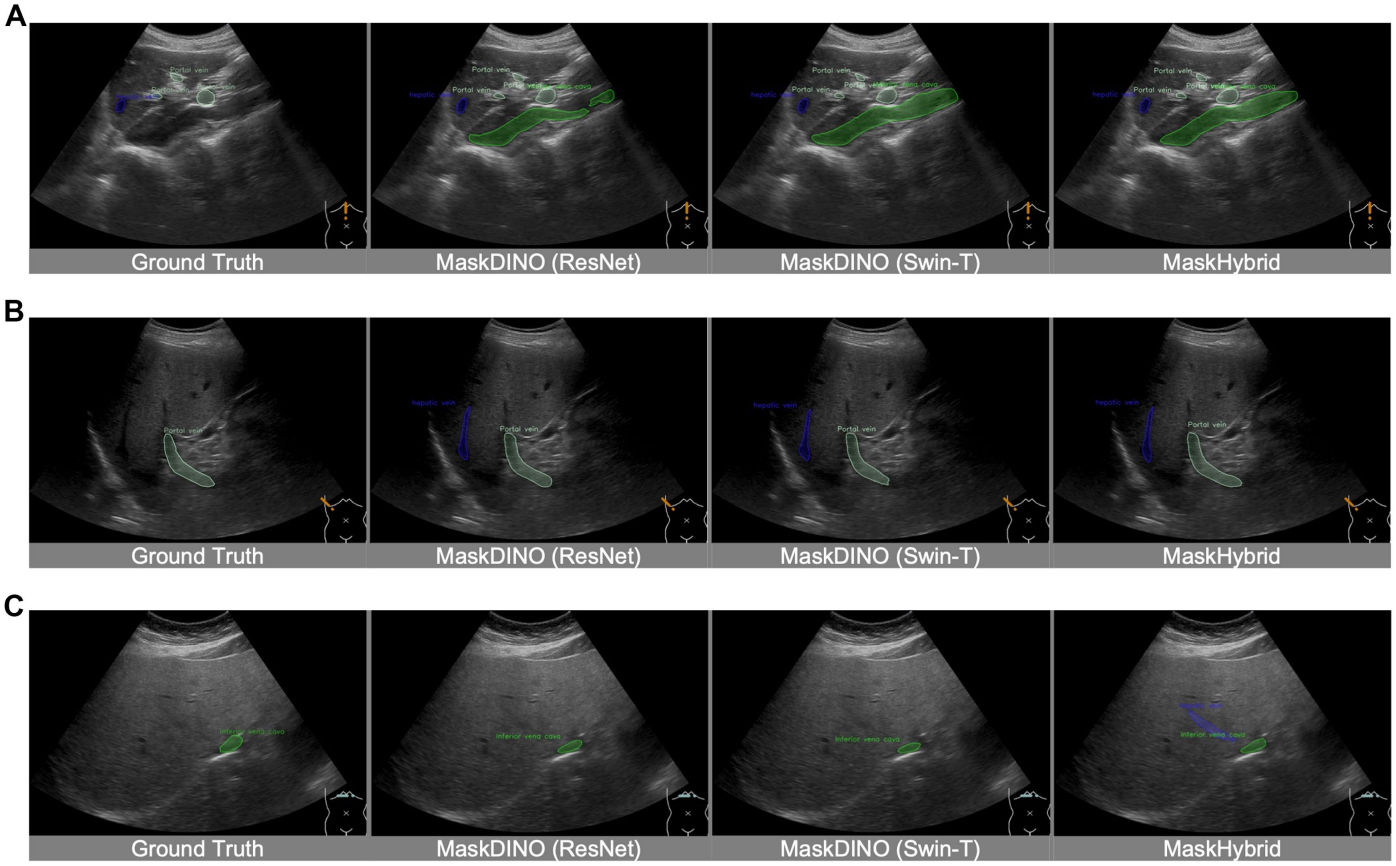
Recognition of unannotated anatomical structures from ground truth. Despite training data limitations, MaskHybrid still effectively identifies anatomical structures in the segmentation of the missing inferior vena cava in **(A)**, the missing hepatic veins in **(B)**, and the missing hepatic veins in **(C)**.

In our retrospective dataset, ultrasound images with mild image conditions, like ascites, were included in the cohort and used for model training, so they could be well recognized. As for images with much intestinal gas were regarded as poor echo windows and excluded at the beginning of the study; therefore, cases with severe gas conditions were out of the scope supported by our AI recognition models. In spite of the fact that the annotations in the training dataset were provided by medical experts, they may not always be perfectly accurate in their annotations. Physicians might overlook certain organs or lesions in US images during the labeling process, such as the inferior vena cava in Figure 6A, the hepatic vein in Figure 6B, and the hepatic vein in Figure 6C. Such training data limitations can restrict training performance. However, our model demonstrates good performance by leveraging long-range dependencies of image features, effectively identifying anatomical structures missed in the ground truth, thereby successfully recognizing the hepatic vein in Figure 6C. Moreover, the model may exhibit errors due to a lack of axis information or incorrect probe orientation. This limitation in spatial recognition may lead to misinterpretations, such as the case in Supplementary Figure 8, where the liver was mistaken for the spleen due to incorrect left–right orientation. Additionally, this study is without external dataset validation.

### 4.4 Inference time

We evaluated model execution time to ensure timely responses during inference for real-time scenarios. As presented in Table 4, the results showed that MaskHybrid exhibited a significantly shorter inference time (0.120 ± 0.013 s) compared to MaskDINO (Swin-T) (0.304 ± 0.019 s) for large-sized AI models of similar size, achieving more than 2.5 times faster. Although MaskDINO (ResNet) achieved the fastest inference time (0.117 ± 0.013 s), this improvement came at the potential cost of segmentation accuracy, as suggested by prior experiments. The slight increase in inference time from MaskHybrid to MaskHybrid with the hybrid encoder (0.122 ± 0.014 s) indicated that incorporating a Mamba-based layer design within the encoder introduced only a marginal computational overhead without significantly compromising efficiency. This finding highlighted the effectiveness of the hybrid architecture in reducing computational complexity while maintaining competitive performance, making our proposed framework well-suited for complex anatomical landmark segmentation tasks in the abdominal US, where accuracy and efficiency are critical in clinical practice.

**Table 4:** Model Inference time of MaskHybrid compared to MaskDINO baselines.

Model	Inference Time (second)
MaskDINO (ResNet)	0.117 ± 0.013
MaskDINO (Swin-T)	0.304 ± 0.019
MaskHybrid	0.120 ± 0.013
MaskHybrid (hybrid encoder)	0.122 ± 0.014

For large-sized AI models of similar size, the MaskHybrid has performed more than 2.5 times faster in inference than MaskDINO (Swin-T).

Overall, the main focus of this pilot study is the recognition and segmentation of anatomical structures and pathological features. Our framework provides comparable execution speed to small-sized segmentation models while offering superior accuracy and visualization compared to common large-sized models, potentially enabling near real-time diagnostic sonography that meets clinical needs. In future work, follow-up studies will evaluate whether the proposed method can distinguish different types of tumors, and a small-scale reader or clinical usability study will be conducted to further evaluate the effectiveness of MaskHybrid in supporting physician interpretation in clinical scenarios.

## 5 Conclusion

In conclusion, the proposed AI-based anatomical recognition framework, MaskHybrid, achieved superior segmentation accuracy and visualization effect for the timely analysis of complex anatomical structures in ultrasound images. Experiments conducted on a retrospective dataset demonstrated the effectiveness and robustness of simultaneously detecting and segmenting multiple abdominal organs and lesions, particularly in challenging anatomical structures. This study is anticipated to facilitate improved diagnostic interpretation of abdominal ultrasound in the near future.

## Data Availability

The data analyzed in this study is subject to the following licenses/restrictions: the ultrasound image dataset and corresponding metadata that support the findings of this study, including annotations and AI models, are not publicly available due to protocol and ethical restrictions of the Institutional Review Board of the Chung Shan Medical University Hospital. However, parts of anonymized data could be made available from the corresponding author upon reasonable request. Requests to access these datasets should be directed to Chi-Chih Wang, bananaudwang@gmail.com.
