# Supplementary material for: AI-assisted anatomical structure recognition and segmentation via mamba-transformer architecture in abdominal ultrasound images
Source: Front Artif Intell. 2025 Jul 23;8:1618607. doi: 10.3389/frai.2025.1618607 (PMC12325247; doi:10.3389/frai.2025.1618607)
Supplement: Supplementary file 1 [file Data_Sheet_1.pdf]

Supplementary Material

Supplementary Material

1 MaskHybrid Framework Details

Major Framework Components. The MaskHybrid framework was based on MaskDINO with the modified backbone, encoder, and decoder, as shown in Supplementary Figures 1-3, to enhance the model performance and visualization effect of anatomical recognition.

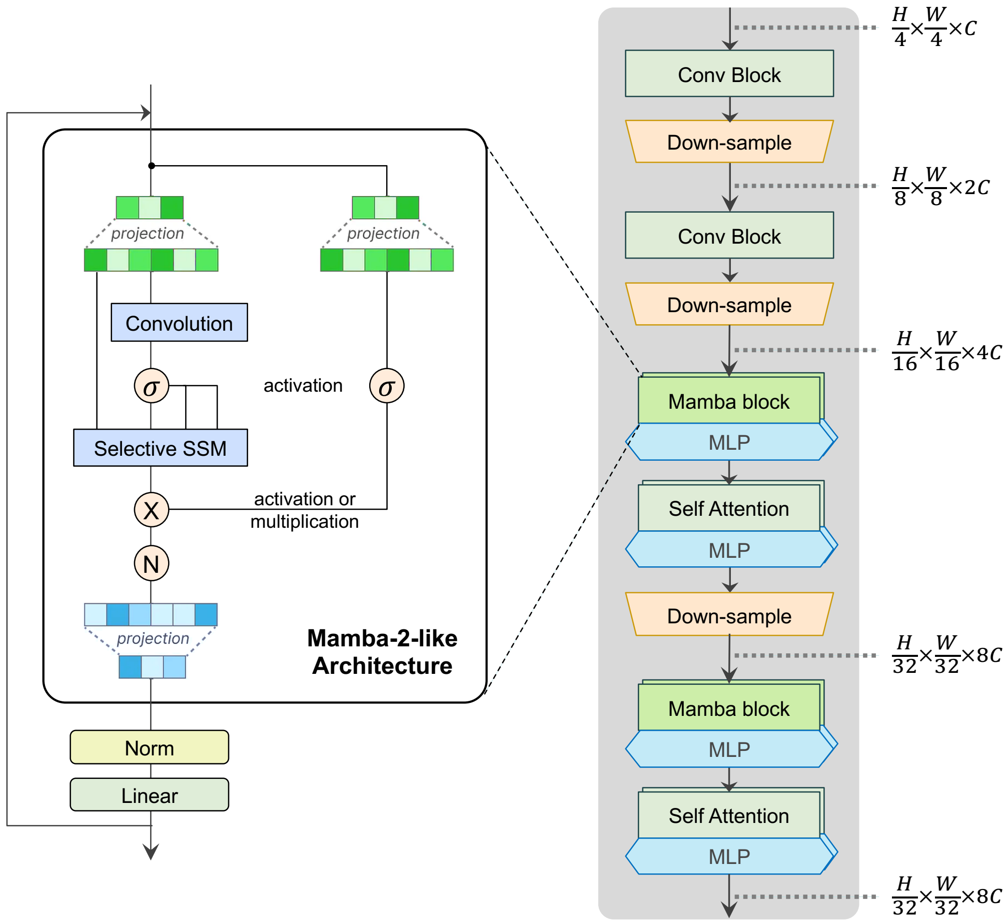

Supplementary Figure 1. Mamba block design inside the mamba-transformer backbone. The global dependencies of ultrasound image features were captured through network layers, and the images were flattened and transformed at different scales for use in MaskHybrid Encoder.

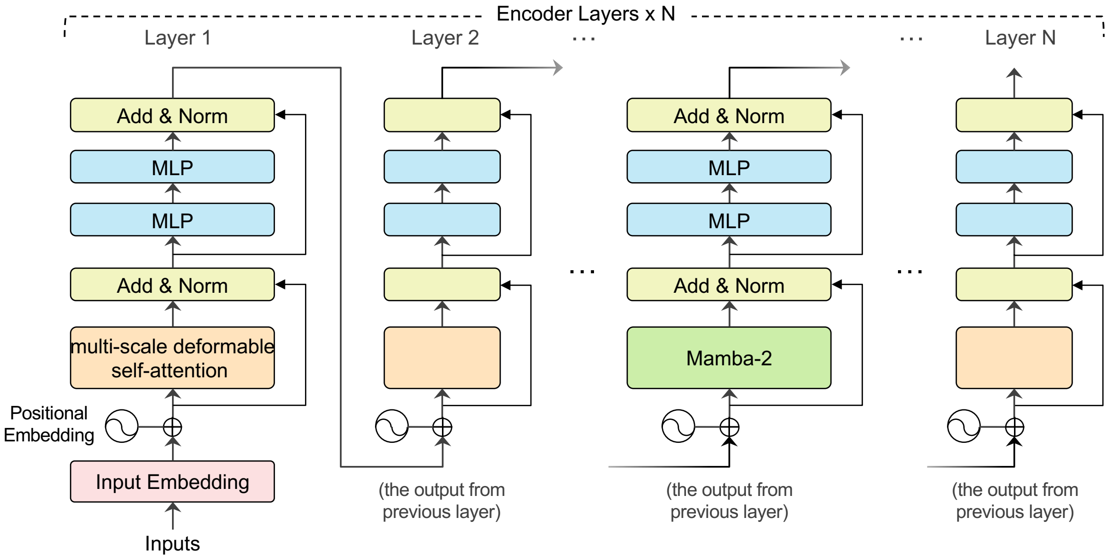

Supplementary Figure 2. Layers' architecture in the MaskHybrid hybrid encoder. The hybrid encoder embedded a Mamba-based layer to enhance the capture of long-range dependencies.

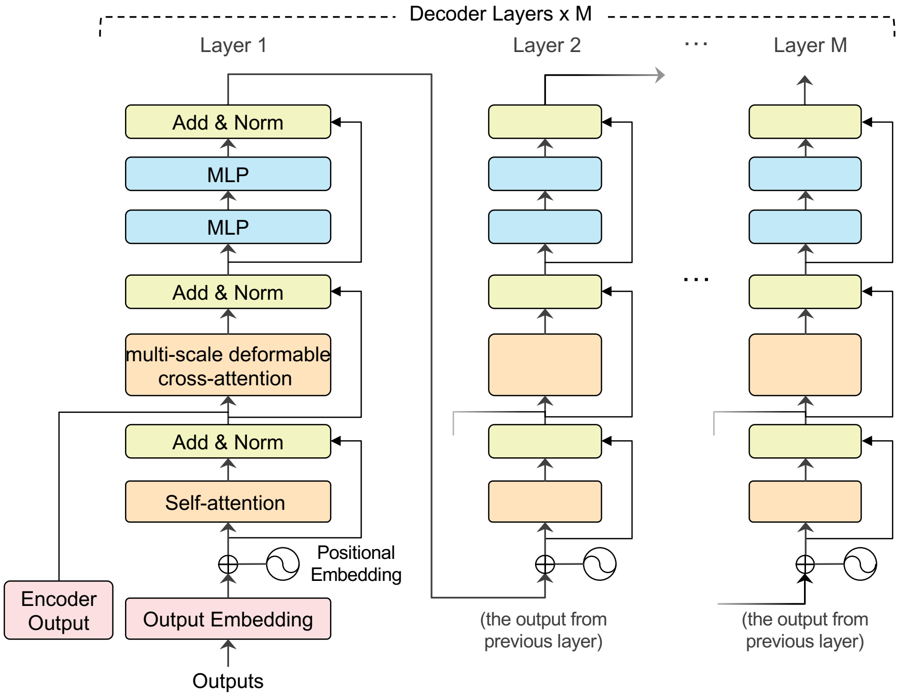

Supplementary Figure 3. Layers' architecture in MaskHybrid decoder. Due to the small number of feature tokens, the decoder layer was retained purely based on the transformer.

2 Model Performance Comparisons

Higher IoU Thresholds. The MaskHybrid design also demonstrated competitive performance than MaskDINO baselines (both Swin-T and ResNet) at AP[.50:.05:.95] thresholds. The interpolated AP when IoU = 0.5, IoU = 0.55, …, IoU = 0.9 and IoU = 0.95 will be calculated respectively, and then average these AP values to get the final mAP.

$$AP[.50:.05:.95] = \frac{1}{10}\sum_{th=\{0.5,0.55,\ldots,0.95\}} AP(IoU_{th} = th)$$

Supplementary Table 1. Image segmentation performance of MaskHybrid under AP[.50:.05:.95] metric compared to MaskDINO baselines with RestNet-50 and Swin Transformer backbones.

Models	dataset	**mAP** (%)	Average Precision (%)								
			Hepatic vein	Inferior vena cava	Portal vein	Gall-bladder	Kidney	Pancreas	Spleen	Hepatic cyst	Tumor
MaskDINO (ResNet)	test	39.71	22.18	41.26	28.84	59.23	75.54	25.86	51.35	**34.25**	18.86
MaskDINO (Swin-T)	test	38.86	20.37	41.65	27.83	58.97	74.42	23.75	50.18	33.46	19.11
MaskHybrid (Mamba-T)	test	**40.94**	**22.43**	**42.66**	**30.29**	**60.68**	**76.75**	**27.45**	**54.51**	34.15	**19.52**

Note. The MaskHybrid framework also demonstrated the overall segmentation performance, reaching 40.94% mAP, surpassing the baselines MaskDINO (Swin-T and ResNet) at higher IoU thresholds from 0.5 to 0.95.

3 Model Visualization Comparisons

Model Consistency for Larger Organs. Generally, all the candidate models have consistent performance in detecting and segmenting larger organs, such as the pancreas and spleen in Supplementary Figure 4; these models have similar visualization outcomes for ultrasound images with significant shadow variations and clear boundaries.

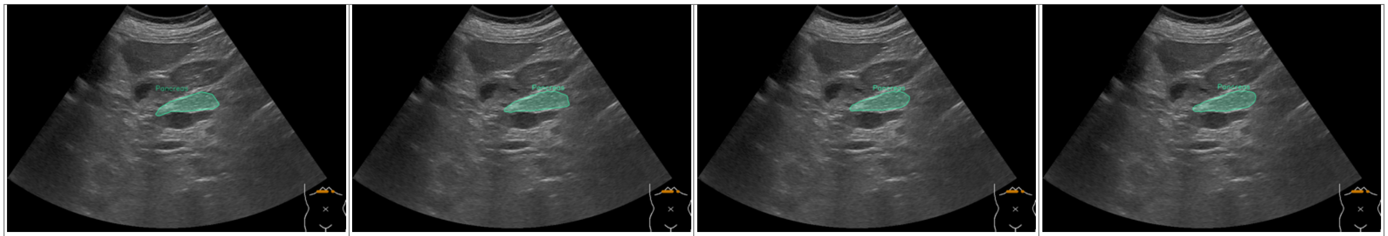

A1) Ground Truth | A2) MaskDINO (ResNet) | A3) MaskDINO (Swin-T) | A4) MaskHybrid

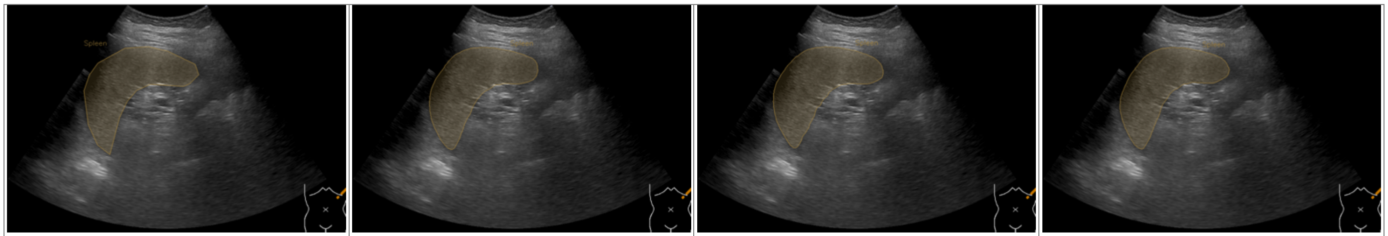

B1) Ground Truth	B2) MaskDINO (ResNet)	B3) MaskDINO (Swin-T)	B4) MaskHybrid

Supplementary Figure 4. Consistency of model performance in anatomical structure recognition. Examples are (A) pancreas and (B) spleen.

Large-sized Models Dominate. Large-sized models, MaskHybrid and MaskDINO (Swin-T), generally provide a deeper understanding of the texture and features of US images, resulting in better visualization of anatomical structure and being less affected by shadowing artifacts, such as the portal vein in Supplementary Figure 5(A) and kidney in Supplementary Figure 5(B).

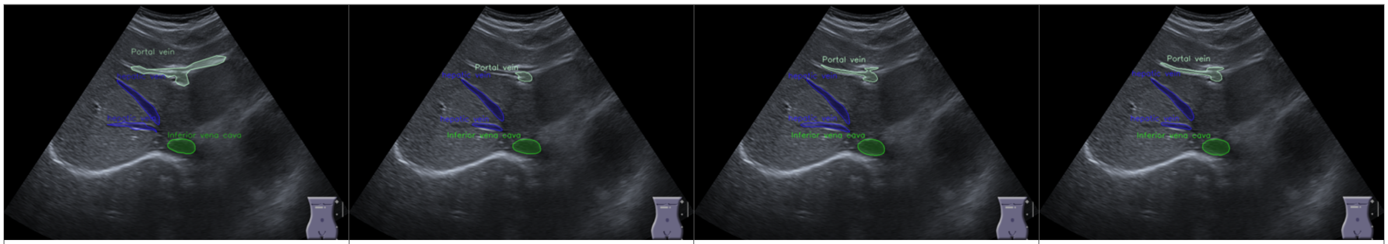

A1) Ground Truth	A2) MaskDINO (ResNet)	A3) MaskDINO (Swin-T)	A4) MaskHybrid

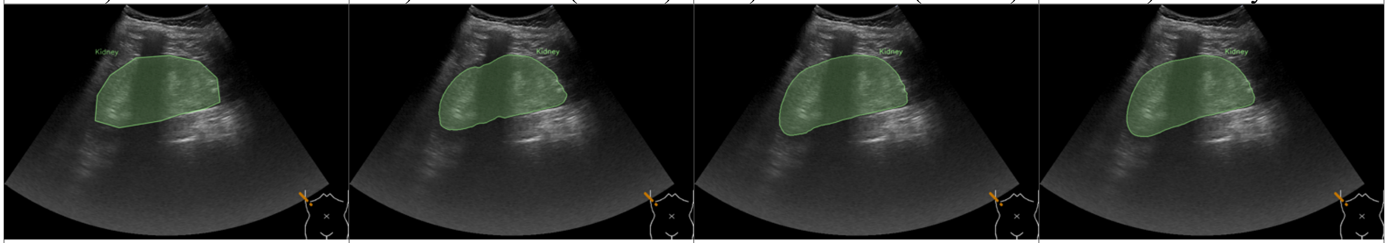

B1) Ground Truth	B2) MaskDINO (ResNet)	B3) MaskDINO (Swin-T)	B4) MaskHybrid

Supplementary Figure 5. Better visualization was provided with large-sized models. Examples are (A) portal vein, hepatic vein, and inferior vena cava, and (B) kidney.

Weak Segmentation for Large Areas in Transformers. The mature transformer-based model, MaskDINO (Swin-T), still has defects in large-area structure recognition and misses the segmentation of partial tumor area, as shown in Supplementary Figure 6(A). Furthermore, MaskHybrid alleviated the issue of overlapping segmented regions in identical anatomical structures, thereby attaining superior overall visualization outcomes, such as the inferior vena cava in Supplementary Figure 6(B).

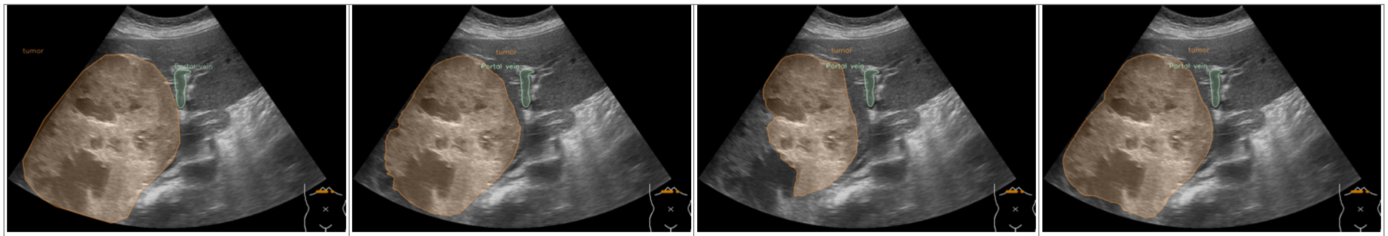

A1) Ground Truth	A2) MaskDINO (ResNet)	A3) MaskDINO (Swin-T)	A4) MaskHybrid

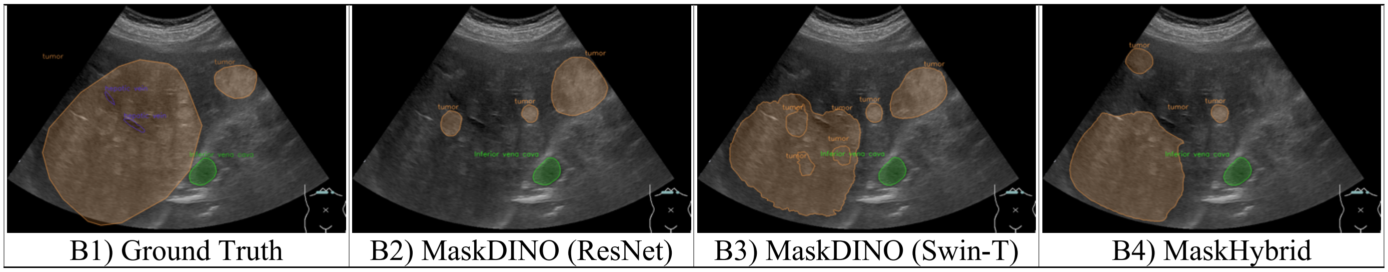

Supplementary Figure 6. Weak Segmentation for Large Areas in Transformer-based models. Examples are (A) tumor and portal vein and (B) tumor and inferior vena cava.

Missing Segmentation with Small-sized Model. Some anatomical structures were poorly represented or even missed in MaskDINO (ResNet), such as the inferior vena cava in Figure S4a, the hepatic cast in Supplementary Figure 7(B), and the portal vein in Supplementary Figure 7(C).

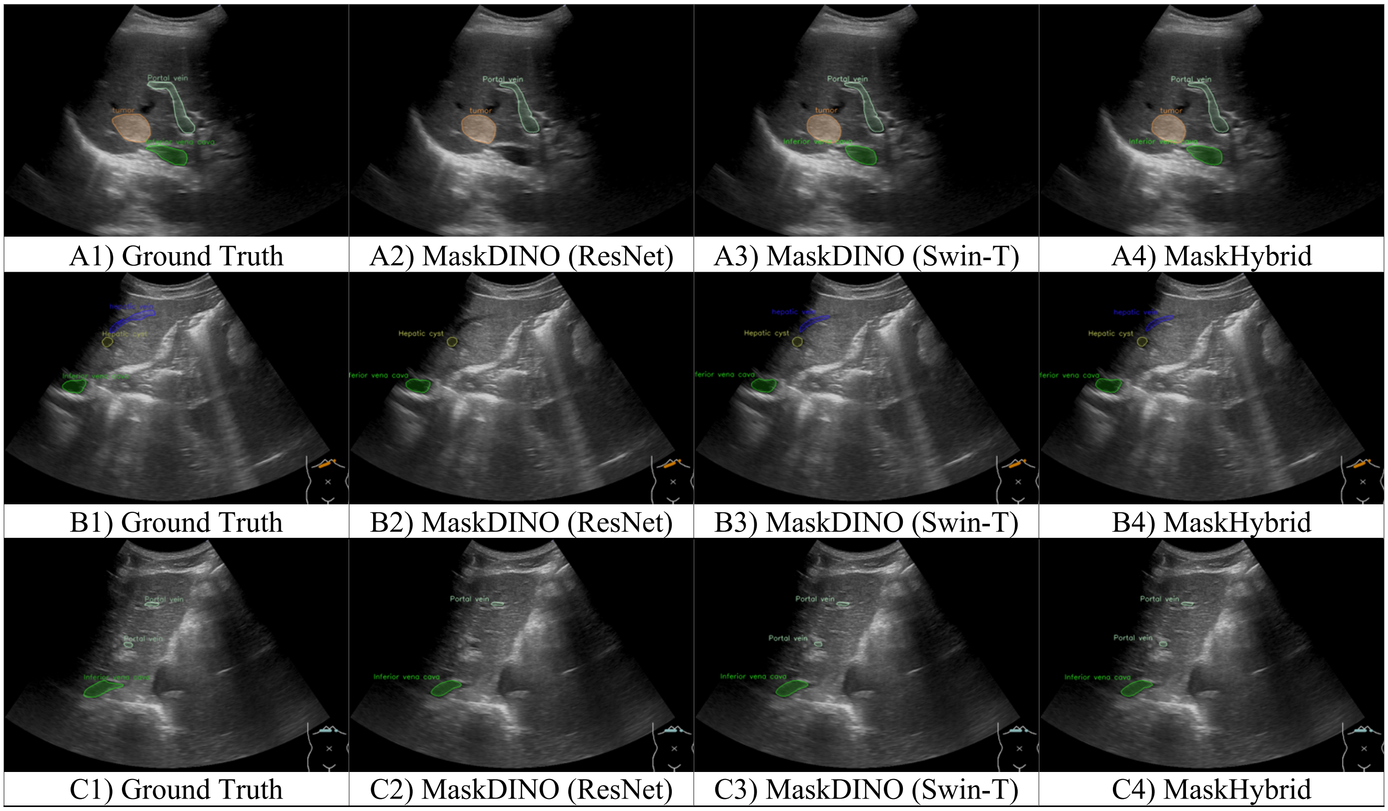

Supplementary Figure 7. Anatomical structure segmentation was missed with small-sized models. Examples are (A) portal vein, tumor, and inferior vena cava; (B) hepatic vein, hepatic cyst, and inferior vena cava; and (C) portal vein and inferior vena cava.

Segmentation Failure in Lack of Orientation. The proposed model exhibits misinterpretation due to incorrect probe orientation, such as the liver in Supplementary Figure 8 was mistaken for the spleen due to incorrect left-right orientation.

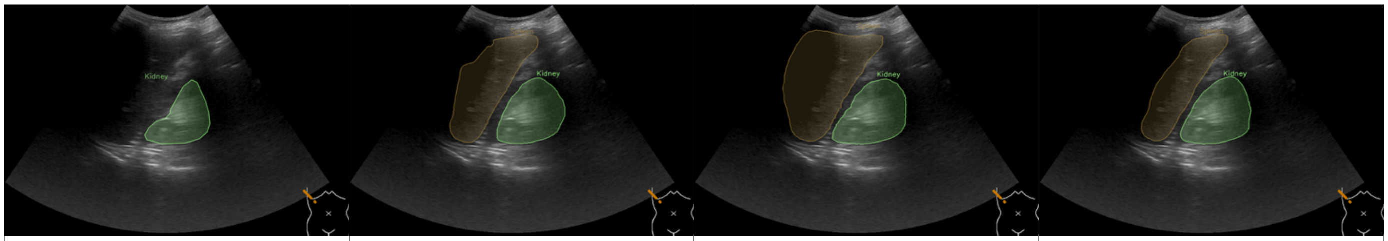

1) Ground Truth	2) MaskDINO (ResNet)	3) MaskDINO (Swin-T)	4) MaskHybrid

Supplementary Figure 8. Segmentation failure due to a lack of orientation information. The example is liver and kidney in the ultrasound image.
